# How the expertise heuristic accelerates decision-making and credibility judgments in social media by means of effort reduction

**DOI:** 10.1371/journal.pone.0264428

**Published:** 2022-03-16

**Authors:** Judith Meinert, Nicole C. Krämer

**Affiliations:** Social Psychology – Media and Communication, University Duisburg-Essen, Duisburg, Germany; Iscte-Instituto Uiversitário de Lisboa, PORTUGAL

## Abstract

Real-time communication, unlimited distribution of information, and the lack of editorial supervision in social media communication aggravate recipients’ credibility evaluations and information selection by what aspects of the source such as expertise have emerged as important anchors for evaluations. It has long been assumed that credibility judgments in social media are specifically guided by heuristics. However, the existing studies merely give indications, for example, based on individuals’ self-report but do not test whether important attributes and prerequisites of heuristic decision-making, such as effort reduction, are present. Against this background, the current study (*N* = 185) analyses by applying a reduced two-alternative choice paradigm whether the relation between the expertise cue and credibility judgments and the choice of information sources is guided by a heuristic, namely the expertise heuristic. Findings indicate that the presence of the expertise cue reduced respondents’ task latencies significantly, although participants’ decision behavior was not independent from additional information. This is discussed in detail with recourse to theoretical conceptualizations of cognitive heuristics.

## Introduction

Social media communication is characterized by floods of information, a lack of gate keepers and quality standards as well as the fast communication flow. However, users tend to utilize social media more and more as main sources for news and political communication [1, 2] which renders the necessity to understand the underlying cognitive mechanisms of credibility judgments and decision-making.

A first attempt into the examination of the cognitive processes underlying online credibility judgments was made by [3] who conducted focus groups. They found recipients to base their evaluations not on content aspects but rather to automatically make use of (unrelated) context information or simple shortcuts. For instance, a well-known company name such as Amazon immediately signals people to be trustworthy just because it is often covered in media and widely known. This (unconscious) usage of mental shortcuts, cognitive heuristics, can be explained by the fact that humans’ cognitive resources are not unlimited [4, 5] and adequate strategies are required to handle large amounts of information as they appear in social media communication.

Sundar [6] attributes the reliance on information bits unrelated to the message to the fact that communication in social media involves many different layers of sources, and meta-informational aspects such as information about the author, the message, the medium, the context and other recipients’ reactions which makes it possible to use simple cues to base judgments on learned and internalized rules. In this vein, he developed a framework, the MAIN model, proposing 29 different heuristics which can be activated by different technology-based cues and further shape individuals’ impressions of whether or not something is credible [6]. Hereby, heuristics are defined as “judgmental rule relevant to credibility evaluations” [6, p. 74] and postulated to connect cues which judgments. For instance, in individuals’ minds exists a “expertise = credibility equation” [6, p. 74] which is learned by experiences and triggered as soon as a relevant marker (so called cue) is received. For this reason, medical information was found to be more likely believed if it comes from a person with an indicated competence such as a doctor degree or a medical organization. This mechanism, however, takes place without even considering the content of the message published by the alleged expert.

In a similar vein, a large body of research observed particularly aspects of the source to play an important role for recipients’ online judgments and decision-making. Source-related information (e.g. reputation indicated by the name) was found to be able to drive users’ selection of online articles [7] and enhanced the confidence in online health information [8, 9]. Furthermore, empirical evidence showed that when the expertise of a source is highlighted (through the name, role or profession), communicated information such as health-related tweets, website articles on media violence and online reviews are perceived as more credible [6, 10–13].

Although, there exists a common understanding among researchers that source cues affect quality assessments in social media communication and the role of cognitive heuristics—as mental strategies for effort reduction—for human judgments and decision-making is highlighted by many scholars, e.g., [3, 6, 14–17], conceptualizations often differ and empirical evidence on cognitive heuristics in the field of online communication is sparse. Apart from focus groups and self-reports [3, 17], which represent an important first approach but are vulnerable for social desirability effects and recognition biases, and outcome-oriented studies [12] manipulating different cues and observing the related outcome (i.e. the judgment), process-related examinations are needed to profoundly address the relation between cues and judgments [18–20] and the question if a heuristic is used or not [21].

Particularly, one crucial aspect of examination is lacking until now. Even though, cognitive heuristics have widely been cited as a strategy to reduce mental effort in social media communication, the effort required to make judgments and decisions in online communication has actually not been empirically investigated to date. As proposed in the framework by [20], the core function of heuristics is effort reduction which can be used as an approach to investigate the relation between cue retrieval and judgment. To measure whether this relation underlies a cognitive heuristic or another more deliberated judgmental strategy, it seems to be a suitable approach to examine if recipients need less effort to arrive at a judgment or a decision. To this end, the current study aims to overcome difficulties of how to measure and further compare and conceptualize heuristics accordingly by defining cognitive heuristics along their cue-wise activation—which means that a single cue is able to activate a related heuristic—[6, 22–24] and effort reduction function [20, 25]. Based on prior findings concerning the predominant role of source expertise in social media communication, e.g., [26] the current study experimentally addressed the expertise heuristic in a reduced two-alternative choice paradigm by investigating the reduction of effort by means of tasks latencies.

## Social media communication and the expertise heuristic

As an ongoing tendency, individuals are using social media channels like Instagram, Facebook, Twitter or YouTube not only for private communication, but also for news consumption [1]. According to the usual customs of social media, news consumption behavior often takes place passively while browsing through the timeline [27] and without an explicit search task. In general, social media communication has been found to be processed in a more peripheral manner [28] compared to traditional media like newspapers, insofar as participants are able to recall fewer content-related arguments and thoughts. This finding can probably be attributed to the fast speed of communication and an almost non-stop exposure to social media content via mobile devices. Regarding humans’ information processing in the realm of social media communication, an increased likelihood of peripheral processing would also imply that cues and indicative stimuli become more relevant and content aspects move into the background. Likewise, social media platforms provide a whole lot of different cues and technological aspects which are able to further guide judgments and evaluations of content quality [6, 23, 29] such as author qualifications, links and pictures within the postings, or likes and shares by other users. The contextual preconditions of social media communication increase the likelihood of relying on simple cues as anchors for credibility evaluations, especially in judgment situations characterized by a high information load which demands large cognitive effort [3, 6].

With the aim of reducing information load, various different cues of social media communication were found to be applied by users for reasoning about information quality [3, 6, 23]. Even if the cues are unrelated to the content or arguments, users, however, did apply them to infer about quality characteristics [30]. In research, there is ample evidence that cues can play a role as anchors for the assessment of credibility in online communication. For example, familiar sources have been shown to be evaluated as more credible [16]. The same applies to an assumed reputation of the source by what automatically the information disseminated by these sources is also accepted as credible [3]. Through these inferences, users save cognitive effort that would be required for an in-depth evaluation of the source *and* the content it promotes [3].

One of the most widely known heuristics postulated in the MAIN model [6] is the expertise heuristic, guided by the underlying assumption “experts’ statements can be trusted” [6, p. 74] [31]. Thereby, solely the indication or perception of a source as experienced increases perceptions of believability. The competency of a source can be gleaned from prior experiences, from experiences shared by other people, from the reputation of the source, or from source credentials, area of work and further aspects [16].

In classical research on persuasion [32, 33], a message’s source was identified as having an influential impact on attitude formation of the audience. Apparently, the source is not only the most salient cue, but recipients have learned to imply the source of information to estimate the value of information [6]. Findings concerning the influence of source credibility can be extended to social media contexts since people adapt and transfer the behavioral pattern of considering the source as an initial step. Accordingly, users have been found to rely on observable aspects of the source while assessing credibility [3, 13]. The impact of source expertise was found to be especially powerful when recipients have less interest or involvement to elaborate extensively on the message and the sender [34].

Prior research provides a lot of evidence for the influence of source cues regarding the expertise, for instance, for the selection of online articles [7, 26], recipients’ confidence in online health information [8, 9] and the believability of online information [3, 11, 12, 35]. Investigating the relative impact of different cues coming along with social media communication, source expertise was obtained to be the most influential aspect for recipients’ credibility evaluations of source and message, compared to likes and shares, pictures and topic involvement [36]. By conducting focus group interviews, [3] evaluated that an already known name of a person, brand or company often implies the attribution of a credible image what they (theoretically) explained with a link between the source cue which triggers a cognitive heuristic and results in a credibility judgment.

However, until now, it is only known that source expertise plays a major role in credibility judgments, but it remains understudied which mechanism underlies the relation between cue and judgment. Even if it can be hypothesized that source cues are able to activate a related heuristic, namely the expertise heuristic, to date empirical evidence for this relation is lacking. By attempting to answer the question of what happens between the cue retrieval of source expertise and credibility judgments and if the relation is guided by the expertise heuristic, we adopted a formalized perspective by relying on the cue-wise activation of heuristics and their effort-reduction function as discriminating factors between heuristics and deliberated judgment strategies.

## Cognitive heuristics for credibility judgments and decision-making

By definition, cognitive heuristics are mental strategies that do not include all available information by what the cognitive load for decisions and judgments is immensely decreased [24]. In this light, cognitive heuristics are simple and efficient shortcuts triggered by a cue, and automatically applied by individuals to protect themselves from cognitive strains and information overload in order to interact efficiently with incoming information [37]. Since people are not aware of these rules which influence their perceptions, and thereby, not able to control these automatic inferences, applying heuristics can lead to valid outcomes, but also to biased judgments [38].

Since cognitive heuristics are supposed to appear in humans’ information processing, especially in situations of higher complexity, overwhelming amounts of information and a lack of high (task-) involvement, social media communication can be deemed as fertile ground for the occurrence of cognitive heuristics. Users of social media are confronted with large amounts of data, real-time communication, high connectivity, and the lack of editorial supervision, which paves the way for information floods and generally complicates evaluations of credibility [23]. Furthermore, an average social media user has to make several decisions, for instance, which information or article to read, which content to judge as reliable, which persons or sources to trust, or which link to follow. This is intensified by the fact that people use social media nearly non-stop via mobile devices so that the information flood (with all resulting tasks) can become unfeasible.

Given that humans’ cognitive resources [4] as well as time and knowledge [39] are limited, cognitive processes such as decision-making and judgments, particularly considering the above mentioned conditions of uncertainty and complexity, come along with the question of how incoming information is processed and integrated. According to dual process models [5, 14], information can be processed through two different routes—the central or the peripheral—which will be taken depending on recipients’ motivation and ability to process information thoroughly. Thus, the likelihood to scrutinize the given information via the central route is increased for recipients with higher interest in the topic or an elaborated thinking style because they strive for valid and truthful attitude formations [40]. In contrast, the peripheral route relates to a simplified processing based on peripheral cues or heuristic rules.

Heuristics can be described as mental operations developed from generalizations and reinforced through experiences [6]. As a consequence, not only the needed resources are reduced, but people are said to be more confident with inferences based on heuristics through an “illusion of validity” [24, p. 11]. That effect can be explained by a simplified cue retrieval and integration in existing knowledge structures because the integration of the cue information has already been established. This simplified fitting of information into pre-existing mental structures is going to be attributed by individuals to a high validity of the cue information, whereas in fact solely the salience and accessibility of an already established connection between a cue and a related inference is enhanced [41].

## Cue-wise activation of heuristics

Heuristics are often criticized as only “loosely characterized” [42, p. 592] without been derived from empirical evidence and utilized to explain nearly any kind of behavior [20, 42] which results in numerous differences regarding their conceptualizations. However, a common understanding exists among researchers that heuristics are triggered by specific cues [14, 20, 24, 43]. Accordingly, the rule concept proposes if-then links between cues and judgments which are connected by “inferential devices” [22, p. 98], a consideration set of rules (either deliberated rules or intuitive heuristics), which are selected based on the requirements of the task [22]. Cues can be represented by various different aspects available in the judgment situation, for instance, by a well-known name of a source.

Following the assumption of the rule concept, the presence of a specific cue can activate a related heuristic which will further lead to a decision or judgment. This process is referred to as cue salience [24]. Deploying this notion to the context of online credibility judgments, cues that are available, accessible, and applicable in online reception situations are said to activate a rule process by which recipients assess credibility based on experienced or socially and culturally learned connections [6, 22, 24, 39].

From a formalized perspective, the underlying structure of cognitive processes like decision-making and judgments can be generally described by cues and related cue values [39] which will be weighted and integrated in information processing [44]. In detail, after a cue is retrieved, a related cue value will be added from memory, the cues are weighted according to individual relevance and lead to a resulting choice or judgment. With regard to decisions between two alternatives, people tend to rely on heuristics when the two options conflict each other in at least one dimension [39]. Consequently, as precondition for the operation of heuristics in decision-making, the cue has to be differentiating between the alternatives [45].

Generally, human decision strategies can be characterized as compensatory or non-compensatory [39, 45–47]. The compensatory fashion is described by taking more than one attribute (i.e. cue) of an alternative and the related value for decision-making and judgment into account. Furthermore, cues are compared and weighted against each other, for instance, a cue with a negative cue value can be compensated by another cue with a positive value. In more detail, first of all, the relevant cues have to be identified, then related cue values are going to be retrieved and evaluated regarding their importance depending on individual ratings and experiences. This is followed by an integration process of all cues into the entire picture. While this is the last step for judgments, for decision-making an additional step is required, picturing the comparison between all present alternatives after which the alternative with the highest value score will be selected [20].

Conversely, non-compensatory strategies which comprise cognitive heuristics focus mainly on one important, discriminating cue [48], and if the related cue value is negative, the alternative is most likely not selected [39]. The “cue-wise” [44, p. 147] activation of heuristics does not require further information and thus, additional information is not integrated into prior knowledge or mental representations but will be ignored [45, 49, 50]. This unconsciously used approach is called attribute substitution [51] and minimizes the cognitive effort of judgments by not including all available information. Strictly speaking, only one cue is used, and not further compared to other cues concerning their values or relevance like it happens for compensatory strategies. Due to the more complex processing and integration of information, the use of compensatory strategies is supposed to require more cognitive effort and thus correspondingly also time than non-compensatory strategies.

In sum, what really draws the line between heuristics and other decision strategies is how information such as cues and their values is integrated in judgments and decision-making [21, 48]. Since people are supposed to stop further processing, evaluation, and information integration if the cue is retrieved which is also characterized by the fact that additional information is ignored, measuring the reduction of effort can be considered as a suitable approach for the investigation whether a non-compensatory strategy like a heuristic was applied. Thereby, it is assumed that the time reduction results from the fact that as soon as the cue is given, all further information is not included and processed so that judgments and decisions are made more quickly.

## Effort reduction as an approach to measure the operation of heuristics

Already dual process models of information processing mentioned the reduction of effort (in cases of a lack of ability, motivation, and interest) as a main driver for peripheral processing [5, 14]. Likewise, cognitive heuristics are defined as strategies used for reducing the cognitive load and complexity of tasks [24]. Correspondingly, [39, p. 91] classified decision and judgments strategies according their “cost and benefits”, whereby costs relate to the effort and benefits describe how accurate the resulting decision is. It can be argued that the outcome and its accuracy perception can influence the experienced usefulness of the chosen strategy. Clearly, more cognitive effort is required for a compensatory comparison of alternatives and their related cues and cue values [39]. Nevertheless, apart from these notions of the influential role of effort reduction for heuristics, it is not specified how the effort is reduced.

Beyond that, [20, p. 208] claim that effort reduction can be regarded as the core function of cognitive heuristics and further be used as “common language to discuss about heuristics” to overcome differences in conceptualizations and domain applications to reach a level of common understanding and comparability. In this vein, they state five different effort reduction principles on which the systematical examination of heuristics can be based to clearly differentiate heuristics from other decision strategies and judgmental rules. The first principle is called *examining fewer cues* and describes that not all cues are considered for decision-making and judgments. Mostly, only one cue will be taken into account, in case of judgments the most important one, in case of decisions, a discriminating one. Even in this process of examining a discriminating attribute, cues are implied subsequently and not at the same time. Reducing effort by considering not all available cues culminates in attribute substitution with solely considering one single cue for judgments and decision-making [51] whereby any further additional information is disregarded. Secondly, *reducing the difficulty associated with retrieving and storing cue values* was proposed for effort reduction. Therefore, individuals particularly use cues which are easy to access due to salience or mental availability and try to avoid complex cues. [19] illustrate this easy-to-access effect with the example that a word’s first letter is always easier to retrieve as the third letter. This mechanism is mirrored in one of the very original heuristics, namely the availability heuristic [24]. For the availability heuristic, humans tend to utilize the ease of retrieval as the basis for a decision or judgment. The third mechanism is called *simplifying the weighting principles for cues*. After receiving a cue, a weight according to the cue value is added. If this effort reduction principle is applied, a weight is added to the cue without any argumentative basis. That can result in every cue receiving the same weight, or the first cue will get the highest weight just because it was first noticed. Overall, cue values are not added according to actual weights, but in a more random fashion instead. Furthermore, *integrating less information* is claimed as a fourth aspect. That means, as soon as an at least acceptable value for a cue is reached, further searching and integrating of information will be stopped. Furthermore, retrieved cues are not compared with and weighted against each other, but evaluated in isolation instead. Thereby, it is a rather intuitive decision between two trade-off cues (or to be precise: cue values) whereby people will tend to select depending on which cue is more relevant and more salient to them. This principle clearly differentiates compensatory from non-compensatory decision strategies. The fifth principle, *examining fewer alternatives* refers to a situation in which individuals can chose how many different options their integrate in their decision. The effort is thereby reduced by simply decreasing the number of options to choose from, for instance, by pairwise comparisons, so that not every option is compared to every other one.

Overall, it is important to emphasize that the proposed effort reduction principles are not explicit and consciously selected by individuals [20, 39]. [20] claim that heuristics are linked to specific effort reduction principles, which additionally indicate that the principles are not exclusive, and a heuristic can make use of several at the same time as it can be seen in the experiments outlined below.

The very first attempts into the experimental investigation of heuristics in decision-making were made by means of the take the best heuristic. In a reduced design four different companies were presented [44], described by six different attributes which were associated with a specific cue value. Participants were instructed to invest a certain amount of inherited money in a company. The cue value was hidden and only revealed if participants clicked on an attribute. Furthermore, at any time only one value was shown. Results revealed that under high time pressure, participants rather used the take the best heuristic. This was figured out because they stopped clicking on the attributes to reveal the hidden cue values as soon as they found a discriminating cue, which fosters one company (they hereinafter chose). Under lower time restriction people stick more to a strategy which sums all positive cue values and thus favor the alternative with the most positive cues. Caused in these findings the authors conclude that differences in required cognitive effort can be related to the use of a heuristic and are depending on time constrains.

In a further experiment by [21] cue valence (with a plus or minus symbol) and the number of additional cues were varied. Findings indicated that the take the best heuristic was outweighed by a simple addition of cues with positive valence. However, it could not be confirmed that additional information like valence and number of additional cues were not considered for the decision which contradicts a non-compensatory decision strategy. Interestingly, participants reported higher confidence evaluations for decisions with positive additional cues which might be explained by an inverse negativity bias [52] through what negative cue values were especially salient and participants tried to avoid them resulting in higher confidence rates for positive cue values.

As the take the best heuristic is highly connected to a search and information integration behavior, experiments on the impact of the recognition heuristic [15] focus more on the presence of one specific cue. Basically, the recognition heuristic states that in decision situations recognized alternatives will be favored, just because of the perceived familiarity and independent from other quality or meta-informational aspects. [53] conducted an experiment using a two-alternatives choice task between a well-known and an unknown brand. Moreover, additional information which was either positive or negative (compared to a control condition with no information) was presented in a learning phase in form of a statement about the brand. It was found that choices were—opposing the assumptions—not independent from the additional information. The recognized alternative was more often selected with positive additional information compared to none and a negative statement. Nevertheless, over all conditions the recognized option was selected more than 50 percent which was interpreted rather as systematic than chance influence of recognition on choices. As a result, authors argued that the nature of the recognition heuristic is probably not non-compensatory but rather compensatory which means that decisions are not exclusively based on one cue.

In contrast, [47] assume that the findings regarding the influence of (positive) additional information might be due to the presentation procedure applied in the study, so they changed the way of how the additional information was presented by using a star rating-system. However, results also showed that decision behavior as well as decision times were not independent from additional information. For the actual decision outcomes, it was revealed that the recognized option was chosen more often when it was accompanied by positive consumer ratings. For the decision latencies, participants were faster in their decision when neutral or positive ratings were present in contrast to negative ones. Overall, it can be stated that the valence of the additional information showed a significant effect on the choices, even if overall choices were driven by the recognition mechanism. However, it has to be considered that the analysis of interaction effects with additional information was solely based on decisions in which participants have correctly selected the well-known product alternative which limits the examination to those individuals who already decided heuristic-wise.

Aiming to find an explanation for the impact of additional information (even if the recognition cue was present), [46] revisited the findings of [47] and investigated the role of the recognition heuristic in comparison with other decision strategies. Basically, [46] states that different persons use different models for deciding between two options. Thus, he tested which strategy was used in a consumer choice task in which participants had to decide between two products. Besides recognition heuristic, equal weight strategy, and weighted additive strategy [39] as well as guess as default option for a random choice were included. Findings indicated that the equal weight strategy accounted best as explanation for the majority of participants’ choices. According to [46], differences in the use of strategies could be generally explained by the influence of personal characteristics which were not addressed to date in the investigation of heuristics.

## Research approach and hypotheses

The effort reduction principles [20] provide a useful approach to experimentally investigate the operation of cognitive heuristics by means of effort reduction. With regard to the expertise heuristic, prior research showed that recipients are used to use the source as an anchor for judgments [3, 6, 7, 26, 32]. Potentially, this can be traced back to the relevance of the source’s status (e.g., in terms of competence) learned from face-to-face interactions [6] and well-established in mental representations about the concept of source expertise. Due to this, the value of the source cue is easy to access for individuals thereby building on the second principle, reducing the difficulty of retrieving cue values.

To measure if the effort is reduced and further if decisions and judgments can be assumed to be based on a heuristic rather than on a compensatory strategy, [25] recommended measuring task latencies as well as task confidence. With a series of computer simulations, it was found that decision times can serve as a valid indicator to identify if an intuitive or deliberated strategy was used, which is probably more valid than the manipulated task time restrictions used by [44] for the examination of heuristics. In line with this, [20] suggest combining latency measures and an outcome analysis as promising for identifying if a cue is actually easy to access and leads to a heuristic-congruent choice or judgment.

Against this background, an experimental study was conducted addressing the expertise heuristic, which is assumed to be activated by the source expertise cue and potentially affects credibility judgments and decision-making. By defining cognitive heuristics based on their cue-wise activation [6, 22–24] and effort reduction function [20, 25] we aimed to overcome difficulties of how to measure, compare and conceptualize heuristics adequately. Following prior experiments investigating the take the best heuristic [21, 44] and recognition heuristic [46, 47, 53], a reduced two-alternative choice paradigm was used. Based on formal descriptions of two alternative information sources, participants were asked to select one and evaluate both afterwards. Following prior attempts of experimental investigations of heuristics [21, 47], the presence of the source expertise cue, the number of additional cues and the valence of additional cues were varied. Therefore, the last two serve the inclusion of further information in the decision situation, so that the hypothesis can be tested that heuristics are working non-compensatory and only the one relevant cue is used for the decision. To account for individual differences and their assumed influence on the reliance of cues according to dual process models of information processing, recipients’ involvement and thinking style preferences were included as potentially influencing factors for the use of the expertise heuristic.

Additionally, age and gender were included as control variables to systematically investigate their potential influence on the dependent variables such as response latencies of the decisions. Indeed, first research [46] suggests that personal characteristics such as age and gender might have an influence on the way people respond to a cue. As additional cues for the experiment, we selected ratings by others, pictures and length (e.g. of a posting or an article) which are relevant aspects in social media communication and are assumed to also being able to influence decisions and judgments. All of them can be theoretically connected with heuristics which could drive users’ inferences [6]. Thus, others’ recommendations are strongly associated with the bandwagon heuristic representing the implicit belief that things recommended by others are of high quality. Pictures are supposed to be more trustworthy than text (“pictures cannot lie”, [6, p. 81]) and length is related to the assumption (“length implies strength” [6, p. 74]). Furthermore, all of them can be considered as influential cues without further context or a message being presented, so that we used these cues as additional cues in the study.

First of all, we attempted to test the salience of the source expertise cue as well as the relation between source expertise and decision-making in the realm of information sources using a reduced two-alternative choice paradigm. Considering previous findings emphasizing the important role of source expertise which was demonstrated to be more important than other cues for credibility judgments, it was assumed:

*H1*: Information sources for which the source expertise cue is present are selected more frequently than information sources for which the ratings of others cue, pictures cue, or articles’ length cue is present.

According to the rule concept [22] cues are connected in form of if-then relations to judgments. As already outlined, several studies provided supporting evidence for this assumption and revealed the influence of source expertise on credibility judgments, e.g., [10, 11, 13]. By transferring these findings to a reduced setting in which source expertise is solely presented as a label, the relation between the cue source expertise and credibility judgments was addressed by the following hypothesis:

*H2*: Information sources for which the source expertise cue is present will be perceived as more credible compared to those without the source expertise cue.

Since intuitive decision strategies like cognitive heuristics require less effort due to a simplified retrieval, processing and integration of cues and related cue values, the question whether the relation between a cue and decision-making is driven by a heuristic, compared to a more deliberated judgment strategy, can be operationalized by investigating if the cognitive effort is reduced [20, 25]. Considering the effort reduction principles stated by [20] the expertise heuristic can be associated with a reduced difficulty of cue retrieval. That means, if the cue (source expertise) is available, salient and easy to access, processing and integration of this information is simplified. Following this approach, by means of decision latencies we aimed to examine whether the source expertise cue activates the expertise heuristic thereby further accelerating the selection of information sources. Therefore, it is assumed:

*H3*: Decision latencies are shorter when the expertise cue is present, and the heuristic is activated as if the expertise cue is absent, and the heuristic is not activated.

Resulting from a confirmation of an already established connection between cue and outcome [24, 25], higher levels of choice confidence (measured through decision satisfaction and certainty) are expected if the cue (source expertise) is present and thereby able to activate the related expertise heuristic. Choice confidence was already found to play a role for the operation of the take the best heuristic [21]. Based on that, the following hypothesis was stated:

*H4*: Decision certainty and decision satisfaction are higher when the expertise cue is present, and the heuristic is activated as if the expertise cue is absent, and the heuristic is not activated.

Heuristics are defined as non-compensatory strategies [39, 45–47], what means they are based on the retrieval of one specific cue [22] whereas all additional information is neglected. Moreover, effort reduction is claimed as core concept differentiating heuristics from deliberated decision strategies [20] and the process is supposed to take place by examining fewer cues and facilitating the effort of retrieving cue values. The latter refers to the use of a cue which is easy to access and perceived as more salient than the other cues due to the ease of retrieval. Whenever a cue is discriminating both alternatives from each other, the alternative with the higher cue value is said to be selected [39] and further cues will not be retrieved and integrated. Thus, the following hypothesis is posited:

*H5*: Additional information such as number and valence of additional cues have no influence on decision latencies, decision certainty and decision satisfaction when the expertise cue is present and the heuristic is activated as if the expertise cue is absent, and the heuristic is not activated.

While prior research on heuristics is limited to decision scenarios, there exists no evidence of how additional information affects credibility judgments based on the operation of a heuristic. Theoretically, it can be argued that the non-compensatory nature of intuitive strategies such as heuristics will lead to attribute substitution for credibility judgments as well whereby individuals base their judgments on one single cue (source expertise) and all other information is ignored [48, 51]. Furthermore, it is stated that the cue-wise activation of heuristics [22, 44] does not require any further information [49]. However, that assumption necessarily requires that the expertise heuristic is triggered and perceived as suitable. Due to a lack of evidence about the role of additional information on credibility judgments, the following research question is posited:

*RQ1*: Does additional information such as number and valence of additional cues influence the impact of expertise on credibility evaluations?

To date, none of the studies addressing heuristics included recipients’ characteristics which, however, potentially determine individuals’ information processing as well as related decisions and judgments. Integrating personal characteristics can be promising to account for differences among participants in their general reliance on cues (and further heuristics triggered by these cues). As stated in dual process models, ability and motivation are decisive for the way of information processing, either central based on arguments or peripheral based on cues. Characteristics like involvement and thinking style preference determine individuals’ level of motivation to scrutinize incoming information [5, 34]. Correspondingly, [54] outlined the importance of individual characteristics such as involvement and thinking styles for a cue to be perceived at all. The cue-salience hypothesis states that the awareness of peripheral cues decreases if topic interest increases. Caused by a decreased accessibility, cues are less salient to recipients [55]. If the source expertise cue is not salient to recipients due to their higher involvement or preference for deliberated thinking, the question arose if the likelihood for the operation of the expertise heuristic is reduced in these cases.

Apart from the findings that female participants were found to base their choices in a probabilistic choice task (e.g., the Linda problem introduced by [56]) more on their intuition compared to male participants [57], gender and age influences on the reliance on heuristics have hardly ever been the subject of research. Nonetheless, to adequately allocate possible conclusions in this respect, we exploratorily incorporated recipients’ age and gender as potential moderators for decision latencies, certainty, and satisfaction as well as credibility assessments. By addressing decision latencies, choice confidence and credibility ratings, the moderating influence of recipients’ characteristics like involvement, thinking style, gender and age is sought to be examined in a comprehensive way. To address these points, the following research questions are stated:

*RQ2*: Do participants´ level of involvement, thinking style preference, gender or age moderate the impact of expertise on response latencies for the decision tasks?
*RQ3*: Do participants´ level of involvement, thinking style preference, gender or age moderate the impact of expertise on certainty and satisfaction with the decision?
*RQ4*: Do participants´ level of involvement, thinking style preference, gender or age moderate the impact of expertise on credibility evaluations of the information sources?

## Method

To investigate the proposed hypotheses, a 2 (presence of the expertise cue: present vs. absent) X 2 (number of additional cues: 1 vs. 2) X 2 (valence of additional cues: positive vs. negative) within-subjects design was employed.

### Design and material

**Fig 1:**
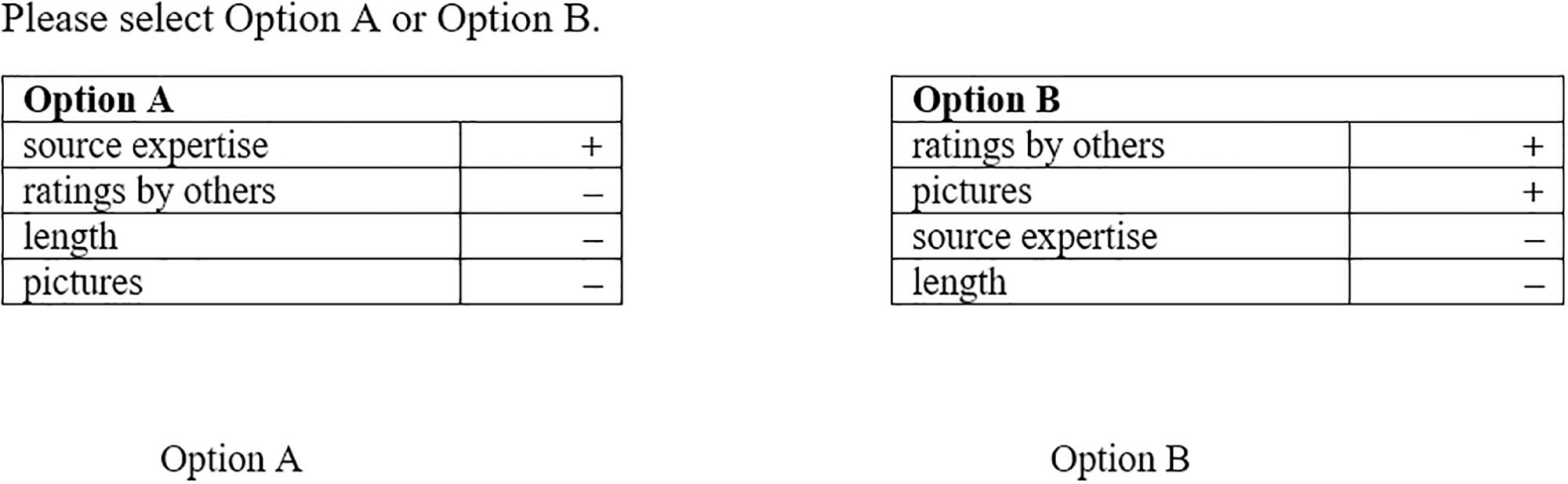
Example for the formal description of information sources with a difference in source expertise (indicated by the cue values).

To avoid effects of prior knowledge as well as interaction effects with the specific content of the messages, a reduced design was used in line with previous work on the systematic investigation of heuristics by [21]. As it was the goal to investigate the impact of different cues on the output (namely the decision) and the reduction of effort measured by means of task latencies differences within the person as a reaction to the different cues present in the decision situation, we used a within-subjects design repeatedly measuring the decisions participants made. Therefore, a two-alternative choice paradigm was applied presenting formal descriptions of two alternative information sources. These descriptions were presented without any content related messages or context information. As describing attributes—apart from the allegedly cue source expertise—aspects that have been highlighted by prior research as highly relevant to social media communication [6, 23] were chosen: the ratings by other users, whether a picture is included and the length of the post. Overall, participants were confronted with six decision tasks for every of the resulting eight factor combinations, so that they performed in sum 48 different decisions between two information source alternatives which were described by four attributes (source expertise, ratings by others, picture, length) and related values indicated by either + symbol or—symbol (see Fig 1 for an example). Beforehand, participants were instructed to imagine that they were scrolling through their Facebook newsfeed and wanted to read a post on a particular topic. They have the choice between two different sources of information, each of which is described by four attributes, which are only formally represented here by the respective word (e.g. source expertise). The alternatives were displayed in a randomized order and the presentation of the attributes as well as the presented position (option A or option B) of the option was counter balanced. The experiment was partly conducted in a lab where up to three participants simultaneously could take part in the study. Additionally, participants were recruited for an online experiment. The procedure of the study was approved by the local ethics committee.

### Procedure

In the beginning, participants were informed about the objective of the study, and that their data will be treated anonymously, will not be passed on to third parties and will be used exclusively for scientific purposes. After providing consent (in written form by clicking on the next button to start the study), participants were instructed that they will be exposed to 48 different decision task (in sum 96 descriptions of information sources) and had to answer some related questions afterwards. In detail, participants were instructed that every decision will be made between two alternative descriptions of information sources, both characterized by four formal attributes with cue values, either indicated by a plus symbol (+) which means that the attribute is present or a minus symbol (-) when the attribute is not present. Furthermore, it was emphasized that participants should strive to make good decisions and that they should not spend more time than necessary for every single decision. Also, they should try to imagine that they are in a realistic situation in which they want to decide which source to choose in order to consume reliable information online.

In line with prior work [21, 44], this was followed by a training phase of the decision task in which participants learned how to conduct the task and select the option they favor. Afterwards, participants performed the 48 decisions each followed by the questions of how certain and how satisfied they are with their choice. In the following, sociodemographic information as well as thinking style preference and involvement were assessed. Then participants were exposed to 24 of the information sources separately and asked to rate their credibility. At the end of the survey participants were informed about the study’s goal and had the opportunity to take part in a prize draw for Amazon vouchers.

### Sample

In total, 187 individuals participated in the experiment (mean age: *M* = 29.06; range: 18–69; *SD* = 10.41), of whom 74 came to the lab and 113 performed the experiment online. For all analyses data from both samples were collapsed since the participation mode (in the lab or online) did not show any systematic influence. Due to extreme values in the processing time of the questionnaire, two participants were excluded from further analysis. The remaining sample of 185 persons had a mean age of 29.06 (range: 18–69; *SD* = 10.41); 100 participants were female and 85 were male. Most of them were students (124 participants), and 40 participants were employees. In general, the sample was highly educated with 33 percent of the participants holding a university degree (61) and 56.2 percent (104) with a high school certificate.

### Measures

#### Decision behavior

For every of the 48 decisions between option A or option B participants were confronted with, we assessed which option they selected.

#### Decision latencies

We measured the response time (in ms) participants needed for every of the 48 decisions between the options A or B (*M* = 5424.69; *SD* = 2315.35). Before the two options were presented, participants saw a white page with the instruction to press ‘M’ on the keyboard if they want to start. When they pressed the button, the two options of information sources were presented side by side and participants could select A or B via the keyboard. The time span between the first click to start the task and the click to select one of the options was assessed in ms.

#### Decision certainty

After every decision it was assessed with a single item on a 11-point Likert scale how certain participants felt with the decision (*M* = 7.88; *SD* = 1.45).

#### Decision satisfaction

In addition, participants were asked to indicate on a 11-point Likert scale how satisfied they were with the decision by a single-item measure (*M* = 8.25; *SD* = 1.48).

#### Credibility

After finishing the decision trials, participants were asked to rate the credibility of the information source descriptions. To preempt fatigue effects, every participant was exposed to randomly selected 24 of the 96 information source descriptions. To assess recipients’ credibility evaluations, the Message Credibility Scale by [58] was used, asking participants on a seven-point Likert scale (1 = describes it very poorly to 7 = describes it very well) how accurate, authentic and trustworthy the source and the hypothetical message is. Since in the current study a potential source of a message had to be rated, the instruction was slightly modified into ‘please rate this information source as well as the hypothetical article’. Additionally, the scale was extended by the items informative, important, qualified, interesting and understandable. Factor analysis (principal component analysis with varimax rotation) yielded a single factor solution (explained variance: 60.99%). Therefore, a mean score was calculated (*M* = 4.78, *SD* = 0.72; *α* = .92), with higher values indicating higher credibility ratings.

#### Involvement

Participants were asked to indicate their involvement in the selection of online information sources. We used the ten-item version of the Personal Involvement Inventory [59] in which items like “important” or “involving” had to be rated on a 7-point semantic differential scale (*M* = 5.08; *SD* = 1.00; α = .91).

#### Thinking style preference

To assess if participants either tend to prefer a deliberated thinking style or an intuitive style in judgment and decision-making situations, we used the inventory for preference for intuition and deliberation (PID) by [60]. Two sub scales measure peoples’ faith in intuition and need for cognition on a 5-point Likert scale (1 = I strongly disagree to 5 = I strongly agree). The sub scale for intuition (PID-I) consists of ten items, e.g, “I like situations in which I have to rely on my intuition” (*M* = 3.47; *SD* = 0.65; α = .81). Need for Cognition is assessed by nine items such as “I think before I act” (*M* = 3.87; *SD* = 0.57; α = .76).

## Results

First, it was tested if the expertise cue worked as intended as the most important cue in decision behavior, which additionally checked the manipulation. To examine *H1*, which posited that information sources for which the source expertise cue is present are selected more frequently than information sources for which the ratings of others cue, the pictures cue or the length cue is present, it was descriptively investigated how often information sources with the different attributes were selected. It has to be considered that every option included four attributes, therefore, the decisions for one attribute reported below are not exclusive, but rather feature the absolute number of choices per attribute.

Among all decisions displaying a difference in the presence of the expertise cue (in total 24 out of 48), in 14 cases the majority of participants chose the option with the expertise cue (indicated by a plus symbol). In sum, 2029 times (out of a total of 4440 if all participants had chosen every time the option with the expertise cue in all conditions with a difference in the presence of the expertise cue) the source with an indicated expertise was chosen, which shows a likelihood of 45.7 percent (see Table 1 for an overview and comparison). The option with the ratings of others cue was chosen 12 times (from total 23 decisions with a difference in the presence of the others’ ratings cue) from the majority of participants. In sum, 1792 times options based on the presence of the ratings of others’ cue (total 4255) were chosen, which relates to a likelihood of 42.1 percent. With regard to conditions representing a difference in the presence of the pictures cue, 9 from 19 times the majority of participants selected the alternative with pictures cue. Altogether, the likelihood for the selection of the option with the picture cue was 32.5 percent with 1144 choices from a total of 3515. From 22 decisions with the length cue, seven were selected by the majority of participants. Thus, the likelihood that the choice was based on the attribute length and the presence of the cue amounted to 20.4 percent with a relation of 829 choices from a total of 4070.

**Table 1:** Likelihood of selection of the information source attributes in percent (random selection likelihood would have been 25 percent).

Information source attributes	Likelihood of selection
source expertise	45.7
ratings by others	42.1
Pictures	32.5
Length	20.4

Afterwards, chi-squared tests were performed to investigate if the number of participants who chose information sources with the expertise cue differs from the number of participants who selected the alternative without the expertise cue. As can be seen in Table 2, the option with the expertise cue was chosen significantly more frequently than the option without the expertise cue among all critical trials (i.e., those with a difference in the presence of the expertise cue). Based on these findings, *H1* can be regarded as confirmed.

**Table 2:** Significant differences (and chi-squared values) in the choices of the information sources with the expertise cue and without the expertise cue (featuring 24 of the 48 decisions).

Decision	information sources with the expertise cue	information sources without the expertise cue	Χ^2^	df	*p*
**1**	136	49	40.91	1	< .001
**2**	139	46	46.75	1	< .001
**3**	149	36	69.02	1	< .001
**4**	155	30	84.46	1	< .001
**5**	150	35	71.49	1	< .001
**6**	157	28	89.60	1	< .001
**7**	156	29	87.18	1	< .001
**8**	145	40	59.60	1	< .001
**9**	144	41	57.35	1	< .001
**10**	147	38	64.22	1	< .001
**11**	138	47	44.76	1	< .001
**12**	148	37	66.60	1	< .001
**25**	111	74	76.95	1	< .001
**26**	109	76	129.87	1	< .001
**27**	105	80	71.99	1	< .001
**28**	152	33	120.01	1	< .001
**29**	170	15	113.65	1	< .001
**30**	150	35	98.51	1	< .001
**31**	167	18	129.87	1	< .001
**32**	165	20	81.78	1	< .001
**33**	160	25	73.96	1	< .001
**34**	170	15	104.44	1	< .001
**35**	154	31	71.49	1	< .001
**36**	151	34	113.65	1	< .001

To examine the impact of the presence of the source expertise cue on credibility judgments of the information sources by assuming that those with the source expertise cue (as indicated by a positive cue value) will be perceived as more credible (*H2*), a paired samples t-test was conducted. Results demonstrated a significant difference in credibility ratings for information sources with the source expertise cue and information sources without the source expertise cue (*T* (47) = 5.09, *p* < .001, d = 2.84). As it can be derived from Table 3 credibility ratings were higher for information sources with the expertise cue. Effect sizes were rather large. In light of these result, *H2* can be confirmed.

**Table 3:** Mean values and standard derivations for the main effect of the presence of the expertise cue on credibility (scale from 1 to 7).

Information sources	*M*	*SD*	*N*
with the expertise cue	5.39	0.38	48
without the expertise cue	4.14	0.76	48

In order to test *H3* claiming that decision latencies are shorter when the expertise cue is present as when the expertise cue is absent, decisions with a difference in the cue value of source expertise (accordingly for one of the information sources the expertise cue was present and for the other one the expertise cue was absent) have been compared to decisions without a difference in the cue value (e.g. in both descriptions of the information sources the cue value of source expertise was equally minus or plus). A paired samples t-test was calculated comparing the decision latencies for decisions with different values for the source expertise cue with the times needed for decisions without a difference in the source expertise cue. Results showed a significant difference in decision latencies (*t* (95) = 11.25, *p* < .001, d = 1.66). Considering calculated mean values (Table 4), choices with a difference in the source expertise cue were generally made faster compared to choices not displaying a difference in the source expertise cue. Effect sizes turned out to be rather large. In sum, *H3* is confirmed.

**Table 4:** Mean values and standard derivations for the main effect of differences in the presence of the expertise cue on decision response time (in ms), decision certainty and decision satisfaction (both scales from 1 to 11).

Expertise	*Decision latencies*	*Decision certainty*	*Decision satisfaction*
Decisions with differences of cue presence	4736.02 (2521.82)	8.18 (1.52)	8.45 (1.50)
Decisions without differences of cue presence	7083.42 (3155.109)	7.54 (1.68)	8.05 (1.77)

To address *H4* and examine if decision certainty and decision satisfaction are higher when the expertise cue is present as when the expertise cue is absent, decisions with different values in the source expertise cue were compared to those without a difference in the presence of the source expertise cue with a paired samples t-test. Thereby, a significant difference in decision certainty was obtained (t (95) = 6.27, p < .001, d = 0.93). Mean values in Table 4 demonstrate that participants were more certain about decisions which featured a difference in the source expertise cue.

A second paired samples t-test addressing differences in decision satisfaction, showed a significant difference in decision satisfaction (t (95) = 4.24, p < .001, d = 0.63). Taken the mean values into account (Table 4), decisions with a difference in the source expertise cue lead to higher satisfaction values. Effect sizes for the effect of the difference in the presence of the source expertise cue on choice certainty and satisfaction were of average heights. Based on these findings, *H4* could be confirmed.

*H5* assumes that additional information (number and valence of additional cues) has no influence on decision times, decision certainty and decision satisfaction when the expertise cue is present and the heuristic is activated as when the expertise cue is absent, and the heuristic is not activated. That means, for decisions with different cue values of the source expertise cue (which is supposed to trigger the heuristic which further guides the selection of the alternative with source expertise), the impact of additional information such as number of additional cues and valence of additional cues is supposed to be eliminated since recipients do not process and include any additional information apart from the cue. To test this hypothesis, three repeated measures ANOVAs (for decision times, decision certainty and decision satisfaction) were calculated with the within subject factors difference of the expertise cue, number of additional cues and valence of additional cues.

### Decision latencies

For response times, an interaction effect of difference of the expertise cue, number of additional cues and valence of additional cues was found (*F* (1,184) = 23.83, *p* < .001, η^2^ = .115). Furthermore, results revealed main effects for the number of additional cues (*F* (1,184) = 33.59, *p* < .001, η^2^ = .155) and the valence of the additional cues (*F* (1,184) = 122.98, *p* < .001, η^2^ = .402). With regard to the mean values (Table 5) the shortest response times emerged for decisions without a difference in the expertise cue with two additional cues, followed by conditions with a difference in the expertise cue and one additional cue and with a difference in the expertise cue and two additional cues. These three conditions have in common that the valence of the additional cues was negative.

**Table 5:** Mean values and standard derivations for the interaction effect of the difference in the presence of the expertise cue, number of additional cues and valence of additional cues on choice response time (in ms).

Expertise difference	Number of additional cues	Valence of additional cues	*M*	*SD*
Decisions with differences of cue presence	2	Negative	4334.04	2043.55
		Positive	5459.06	3230.67
	1	Negative	3991.37	2575.01
		Positive	5159.61	3888.14
Decisions without differences of cue presence	2	Negative	3457.82	1964.85
		Positive	6756.96	3175.84
	1	Negative	6648.89	5208.65
		Positive	7589.77	3913.80

### Decision certainty

A second repeated measures ANOVA with the within-subject factors difference in expertise cue, number of additional cues and valence of additional cues, revealed an interaction between expertise cue difference, cue number and cue valence (*F* (1,184) = 58.24, *p* < .001, η^2^ = .242). In addition, for number of cues (*F* (1,184) = 39.30, *p* < .001, η^2^ = .178) and valence of the additional cues (*F* (1,184) = 36.80, *p* < .001, η^2^ = .168) main effects were observed. Participants assessed to be most certain after decisions without a difference in the expertise cue, two additional cues and positive additional cues, followed by decisions displaying a difference in the expertise cue, one additional cue and positive additional cues (Table 6).

**Table 6:** Mean values and standard derivations for the interaction effect of the difference in the presence of the expertise cue, number of additional cues and valence of additional cues on decision certainty (scale from 1 to 11).

Expertise difference	Number of additional cues	Valence of additional cues	*M*	*SD*
Decisions with differences of cue presence	2	Negative	7.91	1.68
		Positive	8.11	1.54
	1	Negative	8.06	1.92
		Positive	8.61	1.49
Decisions without differences of cue presence	2	Negative	7.50	1.60
		Positive	8.91	1.59
	1	Negative	7.15	2.18
		Positive	6.76	3.03

### Decision satisfaction

To address differences in decision satisfaction, a repeated measures ANOVA was conducted with the within-subject factors difference in expertise cue presence, number of additional cues and valence of additional cues. For expertise difference, cue number and cue valence an interaction effect was found (*F* (1,184) = 56.55, *p* < .001, η^2^ = .237). In addition, the number of additional cues (*F* (1,184) = 46.61, *p* < .001, η^2^ = .204) and the valence of the additional cues (*F* (1,184) = 12.98, *p* < .001, η^2^ = .067) showed a main effect on decision satisfaction. Mean values (Table 7) indicate that participants’ satisfaction reached the highest values after decisions without a difference in the expertise cue, two additional cues and positive additional cues and decisions with a difference in the expertise cue, one additional cue and positive additional cues. Due to the fact that the impact of additional information such as number of cues and valence of the additional cues on decision latencies, decision certainty and decision satisfaction was not systematically diminished in decisions which differed in the presence of the source expertise cue, *H5* has to be rejected.

**Table 7:** Mean values and standard derivations for the interaction effect of the difference in the presence of the expertise cue, number of additional cues and valence of additional cues on decision satisfaction (scale from 1 to 11).

Expertise difference	Number of additional cues	Valence of additional cues	*M*	*SD*
Decisions with differences in cue presence	2	Negative	8.18	1.70
		Positive	8.31	1.49
	1	Negative	8.44	1.78
		Positive	8.82	1.54
Decisions without differences in cue presence	2	Negative	8.30	1.89
		Positive	9.17	1.49
	1	Negative	7.58	2.23
		Positive	7.13	2.97

To evaluate *RQ1* asking if additional information such as the number of additional cues and the valence of additional cues influences the impact of the cue source expertise on credibility evaluations, an univariate ANOVA was performed with the difference in the presence of the expertise cue, cue number and valence as fixed factors and credibility as dependent variable. Results showed a main effect for cue valence (*F* (1, 95) = 61.13; *p* < .001; η^2^ = .394), but no effect for number of cues and no further interaction effects with the expertise cue. In Table 8 mean values are displayed which revealed that information sources with positive cue values were rated as more credible compared to options with mostly negative cue values. Effect sizes were rather large.

**Table 8:** Mean values and standard derivations for the main effect of cue valence on credibility (scale from 1 to 7).

Cue valence	*M*	*SD*	*N*
Negative	4.29	0.70	48
Positive	5.24	0.47	48

To address *RQ2* which addresses moderating effects of participants’ level of involvement, faith in intuition, need for cognition, gender and age on the impact of the expertise cue on decision latencies, moderation analyses were conducted using PROCESS macro. It was found that the impact of the expertise cue on decision latencies was neither moderated by involvement (b = 145.78, 95% CI [-536.018, 827.573], *t* = .422, *p* = .674), faith in intuition (b = 17.46, 95% CI [-1045.330, 1080.243], *t* = .032, *p* = .974), need for cognition (b = 327.14, 95% CI [-869.218, 1523.506], *t* = .540, *p* = .590), gender (b = 992.37, 95% CI [-356.602, 2341.345], *t* = 1.452, *p* = .148) nor age (b = 6.764, 95% CI [-53.873, 72.401], *t* = .203, *p* = .839).

To answer *RQ3* which asks if recipients’ involvement, preference for intuition, need for cognition, age or gender moderate the impact of the expertise cue on decision certainty and satisfaction, moderation analyses (PROCESS) were performed. For the impact of the expertise cue on decision certainty results reveal that none of the moderators involvement (b = -.259, 95% CI [-.681, .163], *t* = -1.21, *p* = .228), faith in intuition (b = -.046, 95% CI [-.704, .613], *t* = -.137, *p* = .891), need for cognition (b = .088, 95% CI [-.657, .833], *t* = .233, *p* = .816), gender (b = .286, 95% CI [-.559, 1.131], *t* = .668, *p* = .505) or age (b = .017, 95% CI [-.024, .058], *t* = .810, *p* = .419) influenced the relation between the expertise cue and decision certainty. For decision satisfaction the moderation analysis (PROCESS) revealed neither involvement (b = -.248, 95% CI [-.679, .182], *t* = -1.138, *p* = .257), faith in intuition (b = -.002, 95% CI [-.671, .667], *t* = -.006, *p* = .996), need for cognition (b = .056, 95% CI [-.810, .697], *t* = -.147, *p* = .883), gender (b = 1.777, 95% CI [-1.042, .687], *t* = -.406, *p* = .686) nor age (b = .031, 95% CI [-.011, .073], *t* = 1.440, *p* = .152) to be a moderator of the relation between the expertise cue and decision satisfaction.

For examining *RQ4* addressing if participants level of involvement, thinking style preference, gender or age moderate the impact of the expertise cue on credibility evaluations of the information sources, moderation analyses (PROCESS) were performed. Results show that the relation between the expertise cue and credibility assessments was not moderated by involvement (b = -.203, 95% CI [-.001, .407], *t* = 1.969, *p* = .059), faith in intuition (b = .338, 95% CI [.023, .653], *t* = .211, *p* = .036), need for cognition (b = .048, 95% CI [-.309, .404], *t* = .265, *p* = .791), gender (b = -.046, 95% CI [-.463, .371], *t* = -.218, *p* = .828) or age (b = .005, 95% CI [-.015, .025], *t* = .482, *p* = .631).

## Discussion

The current study sought to investigate the impact of the presence or absence of the cue source expertise by addressing decision latencies in the context of the selection of information sources. This was based on the rationale that shorter response times can be taken as an indicator for effort reduction coming along with the use of an intuitive, heuristic strategy such as the expertise heuristic when recipients are exposed to a two-alternatives decision situation and the expertise cue is present.

Moreover, it was addressed whether characteristics like involvement, need for cognition or preference for intuition (as indicators for participants’ processing style) as well as gender and age impact decision latencies, choice confidence and credibility evaluations.

First, results confirmed the availability, accessibility and applicability of the expertise cue [6, 43] as alternatives with source expertise cue were chosen more often than alternatives with the other attributes and positive cue values like ratings of other users, pictures or length. Thus, the attribute source expertise was taken as the most important cue for selecting an information source which supports previous findings about the role of source competence [6, 14, 33], e.g. for the selection of online articles [7, 26]. Information sources with source expertise (represented by a positive cue value) were perceived as more credible than those without source expertise (represented by a negative cue value). This finding contributes to previous studies on the important role of source expertise for credibility evaluations, e.g., [10, 11, 13] and further proved the cue source expertise to be influential for decision-making *and* judgments in the realm of information sources, even in a quite reduced design where no further information about the kind of expertise is provided.

The current experiment examined that decision latencies were significantly shorter if the two alternatives differed in the cue value of the expertise cue compared to those decision trials without a difference in the presence of the expertise cue. In detail, participants were faster in selecting one of the information sources when one alternative had a positive cue value for the cue source expertise and the other a negative one compared to choices in which both alternatives had either positive cue values or negative cue values for expertise through what individuals needed more time to choose one of the options. Transferring these findings to the notion of effort reduction as core function of heuristics [24, 25] and potential indicator for the operation of a heuristic [20], it can be argued that in the current study the cue source expertise triggered the expertise heuristics. Reduced decision latencies for decisions in which one alternative was characterized by the presence of the expertise cue provide evidence to this assumption. Basically, individuals saved time when the alternatives were differentiated by the expertise cue (as being either present or absent). Furthermore, this observed tendency conforms to theoretical statements on heuristics referring to reduced cognitive demands [24] and the differentiating function of cues which trigger a related heuristic in humans decision-making under time pressure or uncertainty [39, 45, 48]. The findings of the current study on reduced decision times extended prior results by [44] who investigated participants’ reliance on heuristics when they were set under time pressure for a decision, to a more real-life scenario without artificially restricting task times (which in fact would never be the case with actual decisions). In this regard, participants of the current study perceived source expertise to be the most important cue and if this cue was present and positive for one of the given information sources, the decision was easier and faster. That appears to happen automatically in the current study without time restrictions. In sum, the diminished task latencies for decisions in which the cue was present and differentiated one alternative from the other, seems to be an allusion that participants indeed applied the expertise heuristic for decision-making. Accordingly, the current study could experimentally demonstrate for the first time that the exploitation of the expertise cue for judgments and decision-making actually reduces mental efforts and thus time, and probably works on the basis of a heuristic as it was already commonly assumed but not yet proven [3, 6, 23].

In a similar vein, also certainty and satisfaction with the choice, in sum choice confidence, was higher after decision trials with a difference in the presence of the cue source expertise compared to those without a difference in cue presence. An explanation to this finding can be derived by the match of input and output which is further characterized by an expectancy consistency [24]. Hence, people choose an information source because source expertise was accompanied by a positive cue value, and a connection between source expertise (input) and decision (output) was already established, so that the actual choice is perceived as confirmation for this relation. This effect can be described as a self-confirming circle, potentially able to further strengthen the connection between cue and decision-making which has to be investigated in future studies. Moreover, an information which is easy to retrieve is perceived as the right solution, just because of its easy retrieval [24]. A further explanatory aspect for the increased confidence values refers to the balance between costs and benefits which means that a choice was made which did not require a lot of effort and seem to lead to an adequate solution [39].

By examining the take the best heuristic, [21] also found an effect of higher confidence ratings when a heuristic was used, but this effect interacted with the valence of the additional cues in the decision task. In detail, in his study participants indicated to be more confident after decisions with positive additional cues. In conformity to [21] the current study also obtained an interaction effect of the presence of the expertise cue with additional information on decision latencies, decision certainty and decision satisfaction. Results revealed that decision times were faster for decision trials with two additional cues (contradicting the cue value of the expertise cue) and negative additional cues than for one additional cue and positive valence of the additional cues. An interaction effect showed additionally that participants made the fastest decisions in conditions without a difference in the presence of the expertise cue, with two additional cues and negative additional cues. These findings heavily contradict the concept of attribute substitution, which states that heuristics are only making use of one discriminating cue [51] and do not integrate additional information. However, effects of additional information were found for take the best heuristic [21] and recognition heuristic [47, 53] as well. Since choices potentially based on these heuristics do not seem to be independent from additional information, the authors stated that these heuristics probably are not non-compensatory but rather compensatory which describes that decisions are not exclusively relying on one cue, for instance recognition. Given that the current results are equally not independent from additional information such as the number of additional cues and the valence of the additional cues, it might be assumed that with regard to the expertise heuristic it also has to be considered that the heuristic underlies a more compensatory nature, at least in the current experiment.

However, with regard to effort reduction, it still can be plausible that participants’ cognitive effort was reduced, even if additional information was not ignored. Considering the effort reduction principles by [20], it can be stated that the principle *examining fewer cues* is not applicable to the current results. Notwithstanding, the principle which was said to be related to the expertise heuristic, *simplifying the retrieval of cue values* can still hold true. Indeed, the descriptive data (see Table 1) indicated that source expertise was the cue which was the easiest to retrieve and thereby guided the resulting decision. Still, additional cues seem to have been processed and integrated, but in a subordinate fashion. Of course, this assumption has to be further investigated in future studies. Even if decision latencies are a promising approach to investigate effort reduction, it is not possible to address if and how cue values were retrieved from memory and integrated in information processing as a whole. Therefore, further studies should integrate recall and recognition tasks to test for the easy-to access assumption of cues. In addition, asking participants to note their thoughts after the decision could put the lens on the way of how information is processed and connected to retrieved cue values from memory. This procedure was successfully applied by [28] to explore differences in information processing after individuals’ exposure to either a social media communication or a newspaper interview.

Similar patterns as for the impact of additional information were found for decision certainty and decision satisfaction in the current study, except the fact that for both positive additional cues lead to higher values. [21] explained the influence of positive additional cues on choice confidence as a kind of supportive evidence participants integrated after the choice to validate it. Even if this additional information is irrelevant, the more information is given and the more positive it is, the more confident individuals are after the decision. This explanation approach might probably account for the findings of the current study concerning the impact of positive irrelevant cues on choice confidence.

Basically, for differentiating between intuitive (namely heuristics) and deliberated strategies most researchers conjecture human information processing to be guided by two independent processes, an intuitive and a deliberated one [19]. In contrast, the HSM [14] postulates that both modi can interplay with each other and happen simultaneously. Based on that, peripheral processes such as the operation of a heuristic can interact with deliberated processes. The attenuation hypothesis [31] claims that especially in ambiguous situations cues tend to interact with other incoming information. Similarly, [61] provided an explanation for the influence of additional information which claims that a heuristic plus evaluation mechanism took place. During this evaluation process, further cues are implied. It can be hypothesized that such a process also accounts for the influence of additional cues in the current study.

Furthermore, the valence of the additional cues turned out to influence credibility assessments, insofar that information sources with positive additional cues were perceived as more credible than sources with negative additional cues. This observed effect can potentially be traced back to participants’ inference the more cues are given (as indicted by positive cue values), the more credible the source is. Another explanation which seems to be plausible refers to a negativity bias that is, events, objects or information with a negative valence are perceived as more salient and arousal-evoking than positive ones [52] and thereby information sources with negative irrelevant cues were rated as less credible by participants.

Concerning the influence of recipients’ characteristics like involvement, thinking style preference, age and gender on decision latencies and choice confidence, we found that none of the personal characteristics did moderate the relation between expertise and decision latencies, decision certainty, satisfaction and credibility ratings. The finding that individuals’ reliance on the expertise cue is independent from further variables such as thinking style and involvement suggests the assumption, that heuristics appear to be existing for (almost) all people in a similar manner—as it was already stated in classical conceptualizations [24]. However, it is still possible, that differences are more likely to appear due to situational motivations or prior experiences and generalizations learned therefrom. Of course, these assumption needs further investigation, for instance situational involvement could be manipulated or primed as is was already found to be an influential predictor for information processing and the reliance on cues [5, 55]. For future studies it should be considered that differences between subjects could probably be explained by prior experiences with information sources. Someone who always relies on the Facebook recommendation of a close friend, probably tend to overestimate the effect of recommendations or ratings by other people. On the other hand, if someone has only good experiences by selecting articles based on their authors, the cue source expertise would be more important.

Even though the current study was theoretically embedded in the context of social media communication by using related attributes such as source expertise and other users’ ratings, the used design was much too formal and simple for a practical transfer into the social media realm or for the inference of practical implications and can rather be understood as a first step into the investigation of heuristics that have relevance for social media communication.

## Limitations

When interpreting our findings, some limitations have to be considered. First, participants were exposed to an artificial scenario where they had to choose between only two information sources. In this setting no further context, no task and no articles or messages were provided. In reality, information sources are selected in interdependence with an article or further information. Due to the artificial design of the chosen reduced two-choice paradigm, the external validity is limited. However, applying a reduced artificial setting and following prior approaches [21, 44] allowed for the controlled systematic investigation of decision-making and task latencies due to the exposure of different cues and cue values and to achieve comparability within the research on heuristic processing to a greater extent. Furthermore, by proceeding in this way it was possible to keep all environmental variables constant and avoid interference from the content of a social media posting. By this, we could avoid interference from one’s own subjective attitude to the topic or the author. Despite this, the artificial and formal setting makes it almost unfeasible to draw direct practical conclusions or implications for practical applications in social media communication, which will require further, more practically oriented, research designs.

It should also be noted that a within design with repeated decisions (even if there were only 48 in the current study) is prone to fatigue or learning effects. However, since we aimed to investigate individuals’ responses to all manifestations of the independent variables, we followed prior research in the area of the investigation of cognitive heuristics [21, 44] by applying a within-subjects design forcing participants to perform several decisions to be able to exclude that they have decided only by chance heuristic-wise between the options A and B.

Furthermore, from a methodological viewpoint, credibility judgments were not connected to time measures which potentially would have provided further insights on the effort reduction for judgments. Likewise, credibility judgments and choice behavior were measured independently, although in reality they are highly related to each other. It can be argued that evaluation processes which could possibly explain the effect of the additional information was evoked due to participants’ perception of being under observation which could have strived the motivation to make as good choices as possible (as it was stated in the instruction).

It can be argued that the cues which were presented in the formal descriptions of the information sources (source expertise, ratings by others, length and pictures) could be perceived and evaluated with a different weight of relevance by different recipients which would give them a different degree of subjectivity and limit their comparability within the decision task. Still, specifically with regard to “source expertise” we believe that the chosen setting with the pure formal description and avoiding the naming of a specific source has prevented the interference of subjective evaluations (see above). Furthermore, prior research on heuristics has shown that assuming that all people apply the specific heuristic as soon as they are confronted with a decision task—referred to as the universal hypothesis—[21], represents a sound way to test whether a heuristic is applied through the presence (vs. the absence) of the associated cue. For the current study, we moreover checked if source expertise worked as intended and found that the options favoring source expertise were selected most frequently (as you can derive from Table 1).

A further limitation can be found in the sample composition: Participants were mainly students, consisted of more women than men, and had an above-average level of education.

## Conclusion

The results of the current study highlight that the source expertise cue is able to impact decision-making and judgments in the realm of information sources. Furthermore, the study provides ample evidence for the assumption that the expertise cue activates heuristic processing which was for the first time experimentally confirmed by the fact that decision latencies were diminished. Recipients needed to invest less cognitive effort for decisions in which the expertise cue was present which serves as a strong indicator for the operation of the expertise heuristic.

However, additional information such as number and valence of additional cues was not completely ignored, but rather integrated in information processing which was mirrored in decision times and decision confidence. In sum, the current study can be regarded as first step into the experimental investigation of heuristics by means of testing whether effort reduction takes place. Nevertheless, future studies must address the role of additional information in more depth to gain a clear picture on how heuristics work regarding the use of cues and the integration of additional cue information in information processing.

## Supporting information

S1 File Stimulus material. (PDF)
