# Supplementary material for: How the expertise heuristic accelerates decision-making and credibility judgments in social media by means of effort reduction
Source: PLoS One. 2022 Mar 16;17(3):e0264428. doi: 10.1371/journal.pone.0264428 (PMC8926242; doi:10.1371/journal.pone.0264428)
Supplement: S1 File — (PDF) [file pone.0264428.s001.pdf]

**Option A**	
source expertise	+
ratings by others	–
length	–
pictures	–

**Option B**	
source expertise	–
ratings by others	+
length	+
pictures	–

**Option A**	
source expertise	–
Length	+
ratings by others	+
pictures	–

**Option B**	
source expertise	+
length	–
ratings by others	–
pictures	–

**Option A**	
source expertise	+
pictures	–
ratings by others	–
Length	–

**Option B**	
source expertise	–
pictures	+
ratings by others	–
length	+

**Option A**	
source expertise	–
pictures	+
ratings by others	–
Length	+

**Option B**	
source expertise	+
pictures	–
ratings by others	–
length	–

**Option A**	
source expertise	+
ratings by others	–
Pictures	–
length	–

**Option B**	
source expertise	–
ratings by others	–
pictures	+
length	+

**Option A**	
source expertise	–
ratings by others	–
length	+
Pictures	+

**Option B**	
source expertise	+
ratings by others	–
length	–
pictures	–

**Option A**	
source expertise	+
pictures	–
ratings by others	+
Length	–

**Option B**	
source expertise	–
pictures	+
ratings by others	+
length	+

**Option A**	
source expertise	–
ratings by others	+
pictures	+
Length	+

**Option B**	
source expertise	+
ratings by others	–
pictures	+
length	–

**Option A**	
source expertise	+
Pictures	+
Length	–
ratings by others	–

**Option B**	
source expertise	–
pictures	+
length	+
ratings by others	+

**Option A**	
source expertise	–
Pictures	+
Length	+
ratings by others	+

**Option B**	
source expertise	+
pictures	+
length	–
ratings by others	–

**Option A**	
source expertise	+
Pictures	–
ratings by others	–
Length	+

**Option B**	
source expertise	–
pictures	+
ratings by others	+
length	+

**Option A**	
source expertise	–
length	+
ratings by others	+
Pictures	+

**Option B**	
source expertise	+
length	–
ratings by others	–
pictures	+

**Option A**	
pictures	–
ratings by others	+
length	–
source expertise	–

**Option B**	
pictures	–
ratings by others	+
length	+
source expertise	–

**Option A**	
length	–
pictures	+
source expertise	+
ratings by others	–

**Option B**	
length	–
pictures	+
source expertise	–
ratings by others	–

**Option A**	
ratings by others	–
source expertise	–
length	+
pictures	–

**Option B**	
ratings by others	+
source expertise	–
length	–
pictures	+

**Option A**	
ratings by others	+
length	–
source expertise	–
pictures	+

**Option B**	
ratings by others	–
length	+
source expertise	–
pictures	–

Option A	
ratings by others	–
source expertise	–
Length	–
pictures	+

Option B	
ratings by others	–
source expertise	–
length	+
pictures	+

Option A	
pictures	–
source expertise	–
ratings by others	+
Length	+

Option B	
pictures	–
source expertise	–
ratings by others	–
length	+

Option A	
length	+
pictures	–
source expertise	+
ratings by others	–

Option B	
length	+
pictures	–
source expertise	+
ratings by others	+

Option A	
ratings by others	–
length	+
pictures	+
source expertise	+

Option B	
ratings by others	–
length	–
pictures	+
source expertise	+

Option A	
length	+
source expertise	+
Pictures	–
ratings by others	–

Option B	
length	+
source expertise	+
pictures	–
ratings by others	+

Option A	
pictures	+
source expertise	+
Length	–
ratings by others	+

Option B	
pictures	+
source expertise	+
length	–
ratings by others	–

Option A	
ratings by others	–
source expertise	+
pictures	–
length	+

Option B	
ratings by others	+
source expertise	+
pictures	+
length	–

Option A	
ratings by others	+
pictures	+
source expertise	+
length	–

Option B	
ratings by others	–
pictures	–
source expertise	+
length	+

Option A	
ratings by others	–
Length	–
pictures	–
source expertise	+

Option B	
ratings by others	+
length	–
pictures	–
source expertise	–

Option A	
ratings by others	–
source expertise	–
pictures	+
Length	–

Option B	
ratings by others	–
source expertise	+
pictures	–
length	–

Option A	
ratings by others	–
pictures	–
source expertise	+
length	–

Option B	
ratings by others	+
pictures	–
source expertise	–
length	–

Option A	
length	–
ratings by others	–
pictures	+
source expertise	–

Option B	
length	–
ratings by others	–
pictures	–
source expertise	+

Option A	
length	–
ratings by others	–
source expertise	+
Pictures	–

Option B	
length	–
ratings by others	–
source expertise	–
pictures	+

Option A	
length	–
source expertise	–
pictures	–
ratings by others	+

Option B	
length	–
source expertise	+
pictures	–
ratings by others	–

Option A	
pictures	+
length	+
ratings by others	+
source expertise	–

Option B	
pictures	+
length	–
ratings by others	+
source expertise	+

Option A	
length	+
source expertise	+
ratings by others	–
pictures	+

Option B	
length	+
source expertise	–
ratings by others	+
pictures	+

**Option A**	
length	+
source expertise	–
ratings by others	+
pictures	+

**Option B**	
length	+
source expertise	+
ratings by others	–
pictures	+

**Option A**	
pictures	–
length	+
source expertise	+
ratings by others	+

**Option B**	
pictures	+
length	+
source expertise	–
ratings by others	+

**Option A**	
pictures	+
source expertise	–
length	+
ratings by others	+

**Option B**	
pictures	+
source expertise	+
length	+
ratings by others	–

**Option A**	
pictures	+
ratings by others	+
source expertise	+
length	–

**Option B**	
pictures	+
ratings by others	+
source expertise	–
length	+

**Option A**	
length	–
ratings by others	+
source expertise	–
Pictures	–

**Option B**	
length	–
ratings by others	+
source expertise	–
pictures	–

**Option A**	
pictures	–
length	+
ratings by others	–
source expertise	–

**Option B**	
pictures	–
length	+
ratings by others	–
source expertise	–

**Option A**	
ratings by others	–
Pictures	+
Length	–
source expertise	–

**Option B**	
ratings by others	–
pictures	–
length	+
source expertise	–

**Option A**	
length	–
ratings by others	–
Pictures	+
source expertise	–

**Option B**	
length	–
ratings by others	+
pictures	–
source expertise	–

Option A	
ratings by others	–
Pictures	+
source expertise	–
Length	–

Option B	
ratings by others	–
pictures	–
source expertise	–
length	+

Option A	
pictures	–
length	–
ratings by others	+
source expertise	–

Option B	
pictures	+
length	–
ratings by others	–
source expertise	–

Option A	
length	+
ratings by others	–
source expertise	+
pictures	+

Option B	
length	+
ratings by others	–
source expertise	+
pictures	+

Option A	
pictures	+
ratings by others	–
Length	+
source expertise	+

Option B	
pictures	+
ratings by others	–
length	+
source expertise	+

Option A	
ratings by others	+
source expertise	+
Pictures	–
Length	+

Option B	
ratings by others	+
source expertise	+
pictures	–
length	+

Option A	
length	+
source expertise	+
ratings by others	–
Pictures	+

Option B	
length	+
source expertise	+
ratings by others	–
pictures	+

Option A	
pictures	+
ratings by others	+
source expertise	+
Length	–

Option B	
pictures	+
ratings by others	+
source expertise	+
length	–

Option A	
ratings by others	+
source expertise	+
Length	+
Pictures	–

Option B	
ratings by others	+
source expertise	+
length	+
pictures	–
